# Associations among subjective cognitive complaints, perceived social support, and medication adherence in patients with psychotic disorders

**DOI:** 10.3389/fpsyt.2026.1762058

**Published:** 2026-08-20

**Authors:** Mohamed Hussein Ramadan Atta, Ahmed Hashem El-Monshed, Nadia Waheed Elzohairy, Ahmed Abdelwahab Ibrahim El-Sayed, Darelglal Ahmed Gassmelseed Abdalla, Mohamed Sayed Abdellatif, Magda Mahmoud Mohammad Elgameel

**Affiliations:** 1 Nursing Department, College of Applied Medical Sciences, Prince Sattam Bin Abdulaziz University, Wadi Addawasir, Saudi Arabia; 2 Department of Nursing, College of Health and Sport Sciences, University of Bahrain, Manama, Bahrain; 3 Department of Psychiatric and Mental Health Nursing, Faculty of Nursing, Mansoura University, Mansoura, Egypt; 4 Faculty of Nursing, Damnhour University, Damanhour, Egypt; 5 Nursing Administration Department, Faculty of Nursing, Alexandria University, Alexandria, Egypt; 6 Department of Nursing Sciences, College of Applied Medical Sciences in Wadi Al-Dawasir, Prince Sattam Bin Abdulaziz University, Alkharj, Saudi Arabia; 7 Department of Psychology, College of Education in Al-Kharj, Prince Sattam Bin Abdulaziz University, Al-Kharj, Saudi Arabia; 8 Department of Nursing, College of Applied Medical Sciences, Prince Sattam Bin Abdulaziz University, Wadi Al-Dawaser, Saudi Arabia

**Keywords:** psychotic disorders, cognitive complaints, medication adherence, social support, sustainability, health, self-assessment, psychiatric care

## Abstract

**Background:**

Medication adherence is essential for the management of psychotic disorders, yet it remains difficult for many patients to sustain over time. In this study, the focus is on subjective cognitive complaints, defined as patients’ self-perceived everyday cognitive difficulties, rather than cognitive biases or objectively measured cognitive deficits. These complaints may be associated with difficulties in remembering medication schedules, organizing treatment routines, and maintaining consistent follow-up. Perceived social support may also be relevant, as supportive family members, friends, and caregivers can provide practical and emotional assistance that helps patients manage treatment demands.

**Objective:**

To examine the associations among subjective cognitive complaints, perceived social support, and medication adherence among patients with psychotic disorders, and to assess whether perceived social support statistically accounts for part of the association between subjective cognitive complaints and medication adherence.

**Methods:**

A cross-sectional study was conducted between July 2024 and January 2025 at the psychiatric outpatient clinic of XX. A total of 202 patients with psychotic disorders were recruited using systematic random sampling. Data were collected using the Self-Assessment Scale of Cognitive Complaints in Schizophrenia, the Brief Medication Adherence Rating Scale, and the Multidimensional Scale of Perceived Social Support. Correlation, regression, mediation, and moderation analyses were conducted to examine the relationships among the study variables.

**Results:**

Subjective cognitive complaints were negatively correlated with medication adherence, *r* = –0.193, *p* = 0.005, and perceived social support, *r* = –0.217, *p* = 0.002. Perceived social support was positively correlated with medication adherence, *r* = 0.244, *p* < 0.001. Regression analysis showed that executive function complaints, *B* = –0.667, *p* = 0.016, and language complaints, *B* = –0.905, *p* = 0.038, were significantly associated with lower medication adherence scores. The indirect-effect analysis showed that perceived social support statistically accounted for part of the association between subjective cognitive complaints and medication adherence, indirect effect *B* = –0.019, bootstrapped 95% CI [–0.035, –0.006]. The direct association between subjective cognitive complaints and medication adherence remained significant, *B* = –0.061, *p* = 0.034. Moderation analysis showed that the interaction between subjective cognitive complaints and perceived social support did not reach statistical significance, *B* = –0.004, *p* = 0.077.

**Conclusion:**

Among patients with psychotic disorders, greater subjective cognitive complaints were associated with lower medication adherence and lower perceived social support, while higher perceived social support was associated with better adherence. Perceived social support statistically accounted for part of the association between subjective cognitive complaints and adherence; however, because of the cross-sectional self-report design, these findings should be interpreted as associative rather than causal. Interventions aimed at improving medication adherence may benefit from combining cognitive compensatory strategies with family and social support enhancement.

## Introduction

Medication adherence remains a major challenge in the treatment of psychotic disorders. Nonadherence rates are commonly reported to range between 40% and 60%, increasing the risk of relapse, rehospitalization, symptom worsening, and higher healthcare costs (1). For many patients, adherence is not simply a matter of remembering to take medication. It is shaped by a complex interaction of illness insight, treatment beliefs, objective cognitive functioning, medication side effects, and the degree of support available in daily life (2, 3). Previous studies have examined the relationship between objectively measured cognitive deficits and medication adherence among patients with psychotic disorders (4). However, less attention has been given to patients’ own appraisal of their everyday cognitive difficulties, referred to as subjective cognitive complaints, although this subjective perception may influence their ability to organize treatment routines, evaluate their need for medication, and maintain adherence over time.

Subjective cognitive complaints refer to patients’ self-reported perception of everyday cognitive difficulties, such as forgetfulness, distractibility, mistakes in attention, problems in planning, or perceived failures in monitoring their own actions. This construct should be distinguished from objectively measured cognitive deficits and from the broader concept of metacognition (5). Objective cognitive deficits reflect performance on formal neuropsychological tests, whereas subjective cognitive complaints capture how patients perceive and evaluate their own cognitive functioning in daily life. Although these constructs may be related, they are not identical. A patient may perform poorly on neuropsychological testing without fully recognizing these difficulties, while another patient may report frequent subjective cognitive complaints even when formal cognitive impairment is less pronounced (6). Therefore, examining subjective cognitive complaints may add clinically relevant information beyond objective cognitive assessment alone.

This distinction is important in psychotic disorders, where cognitive difficulties are common and may affect treatment behavior in practical and psychological ways. Patients who perceive frequent subjective cognitive complaints may experience difficulty remembering medication schedules, following treatment instructions, organizing appointments, or maintaining consistent routines. At the same time, self-perceived cognitive difficulties may affect confidence in self-management and increase uncertainty about treatment decisions. For example, a patient who frequently forgets daily tasks or feels unable to organize information may be less able to maintain regular medication use without external support. Conversely, patients who recognize their subjective cognitive complaints may benefit from compensatory strategies, psychoeducation, and structured support to improve adherence. Thus, subjective cognitive complaints may be relevant not because they are identical to objective cognitive deficits, but because they reflect how patients experience their own cognitive functioning in real-world treatment contexts (7, 8).

Social and family support are also important contributors to medication adherence among individuals with psychotic disorders. Supportive relationships may provide emotional reassurance, practical help, reminders to take medication, assistance with appointments, and encouragement to remain engaged in treatment (9, 10). In contrast, low perceived support has been associated with poorer adherence and a higher risk of relapse (11, 12). Social support may be especially important for patients who report greater subjective cognitive complaints, as supportive family members or caregivers can help compensate for forgetfulness, improve medication routines, clarify treatment instructions, and reduce the burden of self-management. In this sense, social support may not only be directly associated with better adherence. Still, it may also help explain why some patients with self-perceived cognitive difficulties are more able than others to sustain medication use.

Although cognitive functioning, subjective cognitive complaints, and social support have each been linked to treatment outcomes, their combined relationship with medication adherence has not been sufficiently examined. In particular, limited evidence is available on whether perceived social and family support helps explain the association between subjective cognitive complaints and sustainable medication adherence among patients with psychotic disorders. This study, therefore, aims to investigate whether subjective cognitive complaints are associated with sustainable medication adherence and whether perceived social and family support plays an explanatory role in this relationship.

These perceived difficulties may be clinically relevant because medication adherence requires patients to remember doses, understand treatment instructions, attend follow-up appointments, and maintain daily routines (7, 8). Previous research has shown that cognitive functioning and treatment-related factors are associated with adherence among patients with psychotic disorders, but subjective cognitive complaints reflect patients’ own appraisal of their daily cognitive functioning rather than objective neuropsychological performance (4, 6). Within this focused framework, perceived social and family support is considered a practical and psychosocial resource that may be associated with better adherence by providing emotional reassurance, medication reminders, assistance with appointments, clarification of treatment instructions, and encouragement to remain engaged in care (9, 10). Low perceived support has also been linked to poorer adherence and relapse risk, suggesting that the social environment may be an important context in which adherence behavior is maintained (11, 12). Therefore, this study examines whether perceived social and family support statistically accounts for part of the association between subjective cognitive complaints and sustainable medication adherence, without assuming causal or directional effects beyond what can be supported by the cross-sectional self-report design (2, 3).

### Study aim

This study aimed to examine the association between self-perceived cognitive Complaints and medication adherence among patients with psychotic disorders, and to assess whether perceived social support mediates this relationship.

## Methods

### Design

This study employed a cross-sectional design and adhered to the Strengthening the Reporting of Observational Studies in Epidemiology (STROBE) guidelines for survey research. Data were collected between July 2024 and January 2025.

### Setting

The study was conducted in the outpatient clinic of XX Hospital for Psychiatric Medicine, which operates under the Ministry of Health and Population. The psychiatric outpatient clinic provides free treatment services to individuals with mental illnesses and substance dependence and operates six days a week (Saturday to Thursday) from 9:00 AM to 1:00 PM.

The services offered at the outpatient clinic include medical examination, diagnosis, prescription of necessary medications, and referrals to inpatient departments when required. Additionally, the clinic plays a crucial role in the follow-up care of previously discharged inpatients. According to hospital outpatient records, the number of patients diagnosed with psychotic illnesses who visit the clinic ranges from 500 to 600 per month. Approximately 75% of these patients attend their appointments regularly.

### Subjects& recruitment sampling

The required sample size was calculated using G*Power (version 3.1.9.7) for a linear multiple regression model. To detect a small-to-moderate effect size (f^2^ = 0.06) with statistical power of 80% and a type I error rate of 0.05, assuming 5 predictors in the regression model, the minimum sample size required was estimated at approximately 200 participants. Accordingly, 202 patients diagnosed with psychotic disorders were recruited for the study. Participants were selected using a systematic random sampling technique with an allocation ratio of 1:3, meaning every third eligible patient was included, ensuring a representative sample while minimizing selection bias.

The recruitment process began with obtaining a daily list of patients attending the psychiatric outpatient clinic, followed by screening for inclusion criteria, which required participants to have a confirmed diagnosis of a psychotic disorder, have never been admitted or discharged at least one month before recruitment, be 18 years or older, and have no comorbid intellectual disability or neurological disorder affecting cognitive function. A random starting point was determined by selecting one patient from the first three eligible individuals, after which every third patient who met the criteria was enrolled. If a selected patient declined participation or failed to meet all inclusion criteria upon further verification, the next eligible patient was included to maintain sampling integrity. This structured and systematic approach ensured an unbiased and representative sample of outpatients with psychotic disorders, enhancing the validity of the study findings.

### Measures of interest

#### The self-assessment scale of cognitive complaints in schizophrenia (SASCCS-Arabic version)

The Arabic version of the SASCCS was utilized to evaluate patients’ perceptions of their cognitive Complaints. The SASCCS is a 21-item self-report instrument originally developed by Johnson et al. (5) and adapted by Haddad et al. (6) for the Arabic population. SASCCS encompasses five cognitive domains: memory (6 items: 1–3, 9–11), attention (5 items: 12–16), executive functions (3 items: 17–19), language (2 items: 20–21), and praxia (5 items: 4–8). Each item is rated on a 5-point Likert scale ranging from 0 (never) to 4 (very often), with the total score reflecting the severity of self-perceived cognitive Complaints.

In terms of reliability, the original scale demonstrated strong internal consistency, with a Cronbach’s alpha of 0.85, indicating a high degree of coherence among the items. Additionally, test-retest reliability was assessed through the intra-class correlation coefficient (ICC = 0.77, p = 0.00), confirming the stability of the scale over time (5). Notably, the Arabic version of the SASCCS, as validated by Haddad et al. (6), exhibited even stronger internal consistency, with a Cronbach’s alpha of 0.911, reinforcing its reliability for use in Arabic-speaking populations.

#### The brief medication adherence scale

The Brief Medication Adherence Scale (BMAS) was utilized to assess participants’ adherence to prescribed medications. Originally developed by Chui et al. (13), the BMAS consists of 10 items, with the first five (Q1–Q5) evaluating attitudes toward medication and the remaining five (Q6–Q10) assessing medication adherence behaviors. Each item is rated on a 10-point scale, yielding a total score ranging from 10 to 100, where higher scores indicate greater adherence.

The BMAS has demonstrated satisfactory psychometric properties. It exhibited a positive and significant correlation with the established Medication Adherence Report Scale (MARS), with a Spearman’s correlation coefficient of 0.56 (p < 0.05), supporting its moderate and acceptable convergent and concurrent validity. Furthermore, test-retest reliability analysis showed a strong correlation between BMAS total scores over time (intraclass correlation coefficient = 0.87, p < 0.05). The internal consistency of the scale, as measured by Cronbach’s alpha, was 0.68, indicating an acceptable level of reliability.

#### Social support scale

The Social Support Scale developed by Sherbourne and Stewart (14) was utilized to assess participants’ perceived social support. This self-report instrument consists of 19 statements categorized into four subscales: tangible support (items 9–12), informational and emotional support (items 1–8), kindness (items 16–18), and positive social interaction (items 13–15). The final item (19) serves as an additional statement. Responses are rated on a 5-point Likert scale ranging from 1 (never) to 5 (always), with total scores ranging from 19 to 95, where higher scores indicate greater perceived social support.

The scale has demonstrated strong psychometric properties. Reliability analysis using Cronbach’s alpha yielded high internal consistency for the subscales: emotional and informational support (0.96), tangible support (0.92), positive social interaction (0.94), kindness (0.91), and the overall scale (0.97). Its validity has been supported through expert evaluations, and previous research among psychotic patients has confirmed its reliability, with a Cronbach’s alpha of 0.97 (15).

In addition to the three tools, the social and clinical data of patients were added from literature (16, 17) to complete the data for all patients.

### Tool validation process

The first tool, the Arabic version of the Self-Assessment Scale of Cognitive Complaints in Schizophrenia (SASCCS), was used directly as it had already been validated in previous research. The second and third tools, the Brief Medication Adherence Scale (BMAS) and the Social Support Scale (SSS), were translated into Arabic using the standard translation and back-translation method to ensure linguistic and conceptual equivalence. A panel of five experts in psychiatric and mental health nursing evaluated the face validity of the translated tools, confirming their clarity and relevance. For content validity, the tools were assessed using the Content Validity Index (CVI). The item-level CVI (I-CVI) ranged from 0.86 to 0.94, and the scale-level CVI (S-CVI/Ave) was 0.91, indicating excellent content validity.

A pilot study was conducted on 20 patients with psychotic disorders (not included in the main study) to test the feasibility and clarity of the tools. The results showed that all items were well understood, and no modifications were required. To assess reliability, Confirmatory Factor Analysis (CFA) was performed, confirming the structural validity of the tools. Internal consistency was evaluated using Cronbach’s alpha coefficient, which demonstrated high reliability: SASCCS (Arabic version) was 0.911, BMAS was 0.72, and SSS was 0.89. These findings indicate that the study tools have good validity and reliability, making them appropriate for assessing cognitive complaints, medication adherence, and social support among patients with psychotic disorders.

### Data collection

Before data collection, all written study materials, including the consent form and questionnaires, were carefully reviewed and checked for clarity and completeness. Upon arrival at the psychiatric outpatient clinic, eligible patients were identified based on the study’s inclusion criteria. After confirming eligibility, patients were approached individually and interviewed, and the interviews lasted approximately 30 to 40 minutes, during which the researcher guided the participant through the questionnaire and clarified any uncertainties. The collected data were reviewed immediately after each session to check for completeness and accuracy.

### Ethical approval

Ethical approval was obtained from the relevant institutional ethics committee before data collection. Administrative permission was also obtained from the responsible authorities at the clinical setting where the study was conducted. The collection and handling of participants’ health-related information were carried out only after obtaining the required approvals and in accordance with institutional regulations for protecting patient data.

Participants were informed about the purpose of the study, the voluntary nature of participation, their right to refuse or withdraw at any time without any effect on their care, and the measures used to protect confidentiality. Written informed consent was obtained from each participant before data collection. Only information relevant to the study objectives was collected.

To maintain confidentiality, no personal identifiers, such as names, medical record numbers, addresses, or telephone numbers, were included in the research dataset. Each participant was assigned a unique code number, and the coding sheet was stored separately from the completed questionnaires/interview forms. Paper-based data were kept in a locked cabinet accessible only to the research team. Electronic data were stored on a password-protected computer/device, and access was limited to authorized members of the research team only. The collected data were used solely for research purposes and were not shared with unauthorized individuals. All findings were reported in aggregate form, ensuring that no participant could be identified from the published results.

### Statistical analysis

The Statistical Package for Social Sciences (SPSS), version 29, was used for data analysis. All the categorical data are summarized as percentages and frequencies. Continuous variables are presented as Mean ± Standard Deviation (SD). The Pearson correlation test was used to correlate continuous parametric data. Linear regression analysis was used to “predict” the value of the dependent variable based upon the values of the independent variables. The ENTER (simultaneous) method was employed, wherein all predictors were entered into the model in a single block without stepwise or automatic selection procedures. Hypothesis testing involved path analysis using the SPSS macro named PROCESS (18). Before conducting linear regression analyses, all underlying assumptions were formally tested. Normality of residuals was confirmed using P-P plots and the Shapiro–Wilk test (*p* > 0.05 for all models). Linearity was assessed via partial regression plots and the Ramsey RESET test (*p* > 0.05). Homoscedasticity was evaluated using the Breusch–Pagan test (*p* > 0.05 for all models). Independence of Complaints was verified with the Durbin–Watson statistic (range 1.8–2.1). Multicollinearity was examined using the Variance Inflation Factor (VIF < 2.5 for all predictors) and tolerance values (>0.40), indicating no significant multicollinearity. All statistical tests were conducted at the alpha level of 0.05 to determine statistical significance. No correction for multiple comparisons was applied because only three theory-driven correlations were tested (H1–H3); all remained significant under Bonferroni (*α* = 0.0167). Bootstrapping was used for mediation.

## Results

**Table 1** outlines the sociodemographic and clinical characteristics of the 202 participants.

**Table 1:** Sociodemographic and clinical characteristics of the study participants (*N*\ =\ 202).

Socio-demographic domains	Socio-demographic factors	*N*	*%*
Age (in Years)	\> 30	53	26.3			
	30 - (\> 50)	130	64.3			
	\≥ 50	19	9.4			
Sex	Male	180	89.1			
	Female	22	10.9			
Marital Status	Single	140	69.3			
	Married	21	10.4			
	Divorced/Widowed	41	20.3			
Level of Education	Basic Education	58	28.7			
	Secondary Education	62	30.7			
	University Education	82	40.6			
Employment Status	Student	62	30.7			
	Working	85	42.1			
	Not Working	55	27.2			
Family Income	Sufficient Income	93	46.1			
	Insufficient Income	109	53.9			
Age (in Years)	60 - (\> 65)	80	39.6			
	65 - (\> 70)	50	24.7			
	\≥ 70	70	34.7			
Living Arrangement	Living with Family Members	179	88.6			
	Living Alone	23	11.4			
Illness Duration	\> 5	70	34.7
	5 - (\> 10)	60	29.7
	\≥ 10	72	35.6
Previous Hospital Admissions	None	22	10.9
	One Admission	100	49.5
	Two or more Admissions	80	39.6
Time Since Last Admission	Less than 1 year	50	24.7
	1 year or more	130	64.3
	Not applicable (never admitted)	22	11
Reason for Discharge	Improvement	130	64.3
	Family Request	38	18.8
	Patient Request	22	10.9
	Escaped	12	5.5
Follow-up Compliance	Compliant	40	19.8
	Non-Compliant	162	80.2
Medication Compliance	Compliant	29	14.3
	Non-Compliant	173	85.7
Social Support Source	Family	111	54.9
	Friends	40	19.8
	Organization	31	15.3
	Other	20	9.9

**Table 2** presents the descriptive statistics of study measures. Cognitive Complaints display a mean of 44.13 (SD = 14.08), reflecting variability across participants. Among cognitive domains, apraxia yields the highest mean (M = 10.76), while language difficulties are the lowest (M = 4.15). Medication adherence has a total mean of 23.64 (SD = 5.8), with adherence behavior (M = 12.74) exceeding attitudes toward medication (M = 10.90). Social support is moderately reported (M = 39.08, SD = 11.16), indicating room for improvement in perceived support levels.

**Table 2:** Descriptive statistics of the study measures (*N* = 202).

Study measures	Minimum	Maximum	M	SD
Cognitive Complaints (Total)	21	77	44.13	14.08
• Memory	6	23	12.30	4.06
• Attention	5	19	10.31	3.66
• Executive Functions	2	11	6.61	2.20
• Language	1	8	4.15	2.04
• Apraxia	5	19	10.76	3.57
Medication Adherence (Total)	15	38	23.64	5.86
• Attitude toward taking medication	6	17	10.90	2.62
• Medication adherence behavior	7	23	12.74	3.94
Social Support (Total)	20	76	39.08	11.16
• Tangible Support	4	16	8.30	2.97
• Information and Emotional Support	8	32	16.45	5.13
• Kindness	3	12	6.03	2.12
• Positive Social Interaction	3	12	6.15	2.59

M, Mean; SD, Standard Deviation.

**Table 3** shows the correlation matrix, revealing significant negative correlations between cognitive Complaints and both medication adherence (*r* = -0.193, *p* = 0.005) and social support (*r* = -0.217, *p* = 0.002). A significant positive correlation exists between social support and medication adherence (*r* = 0.244, *p* < 0.001), highlighting its potential role as a protective factor against cognitive Complaints impacts.

**Table 3:** Correlation analysis between the study measures (*N* = 202).

Study variables		(1)	(2)	(3)	(4)	(5)	(6)	(7)	(8)	(9)	(10)	(11)	(12)	(13)	(14)
Cognitive Complaints (1)	*r*	1													
	*P*														
Memory (2)	*r*	.929^**^	1												
	*P*	<0.001													
Attention (3)	*r*	.911^**^	.777^**^	1											
	*P*	<0.001	<0.001												
Executive Functions (4)	*r*	.827^**^	.724^**^	.636^**^	1										
	*P*	<0.001	<0.001	<0.001											
Language (5)	*r*	.875^**^	.863^**^	.761^**^	.613^**^	1									
	*P*	<0.001	<0.001	<0.001	<0.001										
Apraxia (6)	*r*	.937^**^	.785^**^	.852^**^	.815^**^	.736^**^	1								
	*P*	<0.001	<0.001	<0.001	<0.001	<0.001									
Medication Adherence (7)	*r*	-.193^**^	-.154^*^	-.176^*^	-.249^**^	-.219^**^	-.125	1							
	*P*	.005	.027	.011	<0.001	.001	.071								
Attitude toward taking medication (8)	*r*	-.183^**^	-.121	-.141^*^	-.301^**^	-.174^*^	-.155^*^	.834^**^	1						
	*P*	.008	.082	.042	<0.001	.012	.025	<0.001							
Medication adherence behavior (9)	*r*	-.165^*^	-.148^*^	-.168^*^	-.170^*^	-.210^**^	-.083	.931^**^	.574^**^	1					
	*P*	.018	.033	.015	.014	.002	.235	<0.001	<0.001						
Social Support (10)	*r*	-.217^**^	-.105	-.257^**^	-.272^**^	-.218^**^	-.179^**^	.244^**^	.269^**^	.184^**^	1				
	*P*	.002	.131	<0.001	<0.001	.002	.010	<0.001	<0.001	.008					
Tangible Support (11)	*r*	-.091	.125	-.226^**^	-.113	-.067	-.161^*^	.102	.117	.074	.716^**^	1			
	*P*	.191	.072	.001	.105	.336	.020	.141	.092	.287	<0.001				
Information and Emotional Support (12)	*r*	-.178^*^	-.032	-.238^**^	-.230^**^	-.196^**^	-.165^*^	.242^**^	.289^**^	.167^*^	.961^**^	.734^**^	1		
	*P*	.010	.646	.001	.001	.004	.017	<0.001	<0.001	.016	<0.001	<0.001			
Kindness (13)	*r*	-.175^*^	-.232^**^	-.129	-.244^**^	-.152^*^	-.056	.165^*^	.186^**^	.121	.657^**^	.093	.511^**^	1	
	*P*	.012	.001	.063	<0.001	.029	.424	.017	.007	.082	<0.001	.180	<0.001		
Positive Social Interaction (14)	*r*	-.233^**^	-.225^**^	-.189^**^	-.282^**^	-.240^**^	-.158^*^	.253^**^	.245^**^	.213^**^	.818^**^	.267^**^	.722^**^	.739^**^	1
	*P*	.001	.001	.006	<0.001	<0.001	.023	<0.001	<0.001	.002	<0.001	<0.001	<0.001	<0.001	

*r=* Pearson correlation.

**Correlation is significant at the 0.01 level (2-tailed).

*Correlation is significant at the 0.05 level (2-tailed).

**Table 4** reports regression analysis findings, explaining 12.6% of the variance in beliefs about medication adherence (*R*^2^ = 0.126). The model is significant (*F*(6, 201) = 4.121, *p* < 0.001). Among predictors, executive function (*B* = -0.667, 95% CI [-1.206, -0.128], *p* = 0.016) and language difficulties (*B* = -0.905, 95% CI [-1.761, -0.049], *p* = 0.038) were significantly associated with lower adherence beliefs. Memory shows a marginally significant positive association (*B* = 0.518, 95% CI [-0.047, 1.083], *p* = 0.072).

**Table 4:** Regression analysis of cognitive complaints and social support predicting beliefs about medication adherence in patients with psychotic disorders.

Predictors	Beliefs about medication adherence						
	Unstandardized coefficients		Standardized coefficients	*t*	*P*	95% CI for the difference	
	*B*	*SE*	*ß*			Lower bound	Upper bound
Constant	23.316	2.229		10.462	.000	18.921	27.710
Memory	.518	.287	.359	1.806	.072	-.047	1.083
Attention	-.036	.194	-.022	-.184	.854	-.418	.347
Executive Functions	-.667	.273	-.251	-2.441	.016	-1.206	-.128
Language	-.905	.434	-.315	-2.086	.038	-1.761	-.049
Information and Emotional Support	-.009	.138	-.008	-.067	.947	-.281	.263
Kindness	.004	.284	.001	.015	.988	-.556	.564
Positive Social Interaction	.424	.286	.188	1.481	.140	-.141	.989

Effect (B), Unstandardized Coefficient; *ß*, standardized coefficient beta; SE, Standard Complaints; *t,* Student t test for linear regression.

95% CI for difference 95% Confidence Interval for Difference.

*R^2^* = 0.126 (*Adj R^2^* = 0.095), *F-*change = 4.121, P<0.001.

Beliefs about Medication Adherence (dependent variable) reflect the total BMAS score (Q1-Q10), combining both attitude and behavior domains.

**Figure 1:**
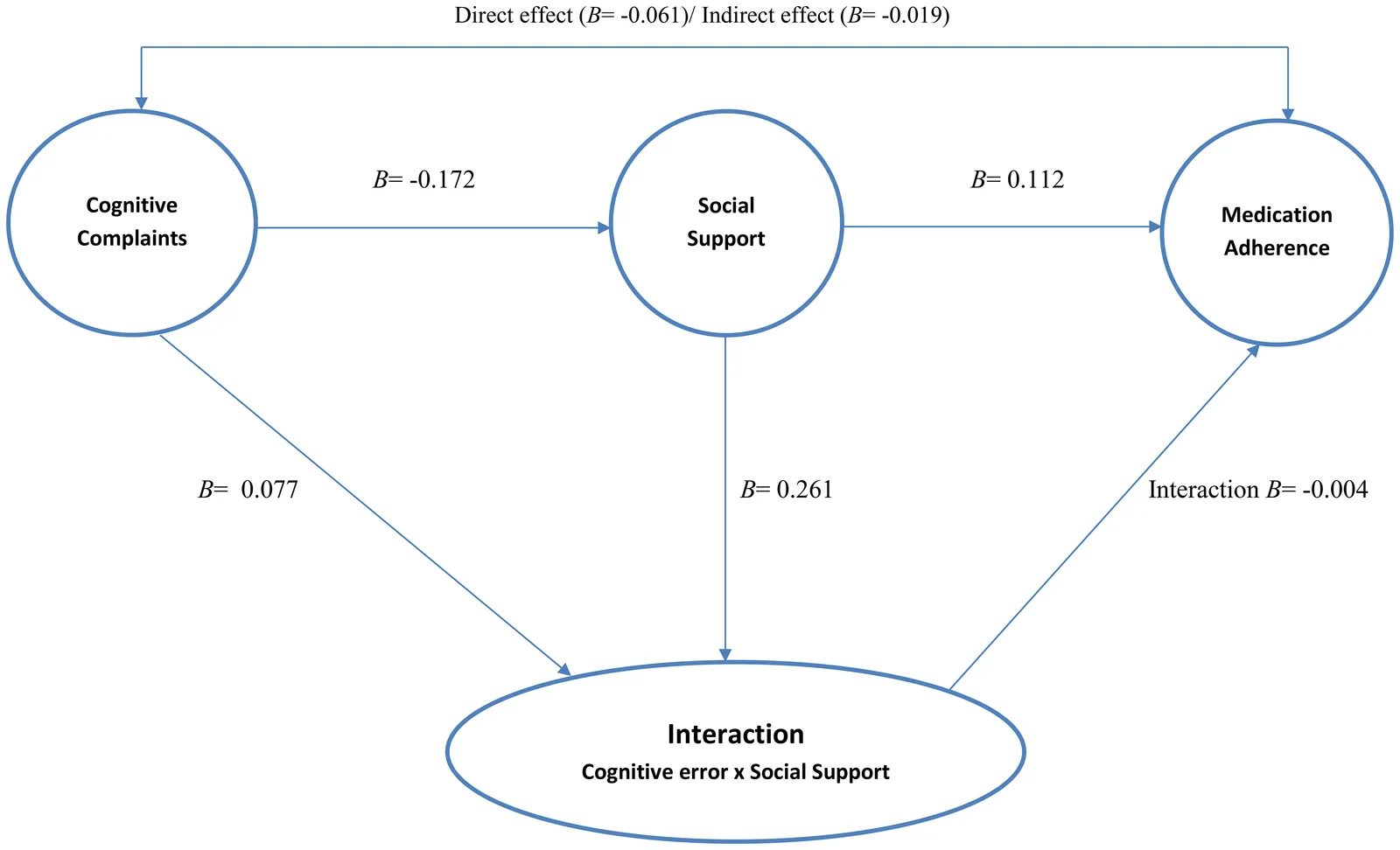
Pathways of mediation and moderation effects of social support on medication adherence to cognitive complaints.

**Table 5** and **Figure 1** detail the mediation analysis, demonstrating that social support partially mediates the relationship between cognitive Complaints and medication adherence beliefs. The total effect is significant (*B* = -0.080, 95% CI [-0.136, -0.024], *p* = 0.005), as is the indirect effect via social support [*B* = -0.019, 95% CI (-0.035, -0.006), *p<0.001*], representing a clinically meaningful mediation that accounts for 23.9% of the total effect. The mediation model explained 8.0% of the variance in adherence beliefs (*R*^2^ = 0.080). In clinical contexts, this suggests that strengthening social support systems may yield tangible benefits in promoting medication adherence.

**Table 5:** Mediation analysis of the role of social support in the relationship between cognitive complaints and beliefs about medication adherence.

Path	Effect (*B*)	*SE*	*t*	*p*	95% CI for the difference	
					Lower bound	Upper bound
CC → SS	-0.172	0.054	-3.189	.0017	-0.278	-0.066
SS → BMA	0.112	0.036	3.095	.0022	0.044	0.183
CC → BMA Direct Effect	-0.061	0.029	-2.139	.0336	-0.118	-0.005
CC → BMA Total Effect	-0.080	0.029	-2.820	.0053	-0.136	-0.024
Indirect Effect CC → SS → BMA	-0.019	0.007	–	–	-0.035	-0.006

CC, Cognitive Complaints; SS, Social Support; BMA Beliefs about Medication Adherence (dependent variable) reflect the total BMAS score (Q1-Q10), combining both attitude and behavior domains.

Effect (B), Unstandardized Coefficient; SE, Standard Complaints; *t=* Student t test for linear regression.

95% CI for difference 95% Confidence Interval for Difference.

Model Summary for Social Support (Mediator, M): R^2^ = 0.047, F(1, 206) = 10.17, p = 0.0017.

Model Summary for Beliefs about Medication Adherence (Y): R^2^ = 0.080, F(2, 205) = 8.93, p = 0.0002.

Bootstrap Confidence Interval for Indirect Effect: Bootstrapped 95% CI: [-0.035, -0.006] (Statistically Significant).

**Table 6** and **Figure 1** present the moderation analysis. The interaction between cognitive Complaints and social support did not reach the conventional level of statistical significance (*B* = –0.004, 95% CI [–0.008, 0.000], *p* = 0.077). The overall model accounted for 9.4% of the variance in adherence beliefs (*R*^2^ = 0.094).

**Table 6:** Moderation Analysis of the Relationship Between Cognitive Complaints and Medication Adherence: The Role of Social Support.

Coefficients	Effect (*B*)	*SE*	*t*	*p*	95% CI for the difference	
					Lower bound	Upper bound
Constant	16.330	3.815	4.281	<0.001	8.808	23.852
Cognitive Complaints	0.077	0.083	0.929	0.354	-0.086	0.239
Social Support	0.261	0.092	2.854	0.005	0.081	0.441
Interaction Term (Cognitive Complaints x Social Support)	-0.004	0.002	-1.777	0.077	-0.008	0.000

Effect (B), Unstandardized Coefficient; SE, Standard Complaints; t, Student t test for linear regression.

Model Summary: R^2^= 0.094, F = 7.070, p(Model)= 0.0002.

Test of Interaction Effect: R^2^ Change 0.0140, F (Interaction)= 3.156, p (Interaction)= 0.077.

Beliefs about Medication Adherence (dependent variable) reflect the total BMAS score (Q1-Q10), combining both attitude and behavior domains. .

**Figure 2:**
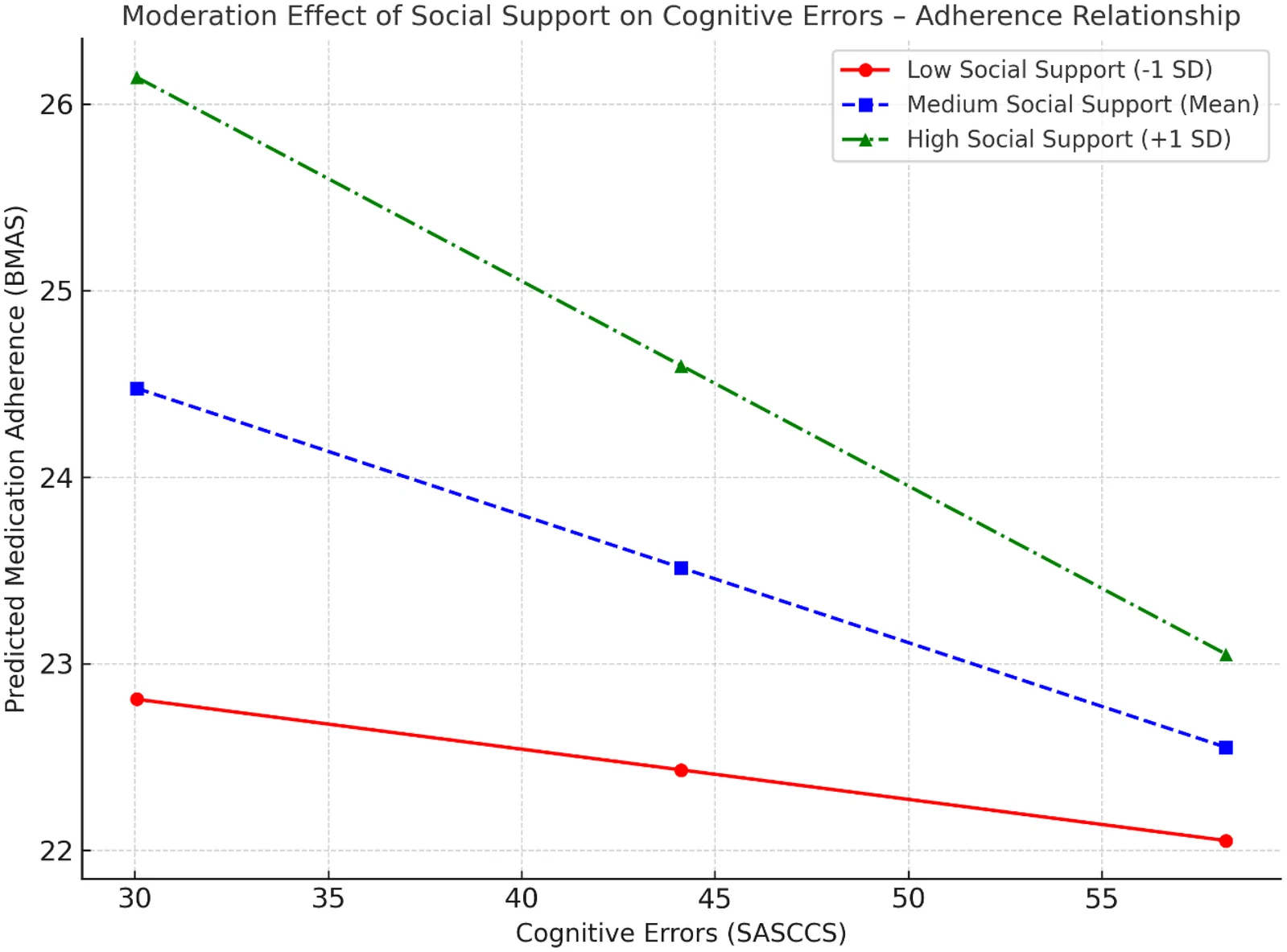
Simple slopes of social support moderating the relationship between cognitive complaints and medication adherence beliefs.

**Figure 2** illustrates the conditional effect of cognitive Complaints on beliefs about medication adherence (BMAS) at low (–1 SD), mean, and high (+1 SD) levels of social support. This figure shows that individuals with high social support tend to have better adherence beliefs at lower cognitive error levels, but this advantage diminishes as cognitive Complaints increase.

## Discussion

This study examined the associations among subjective cognitive complaints, perceived social and family support, and medication adherence among patients with psychotic disorders. Overall, the findings suggest that medication adherence behavior was higher than attitude toward taking medication. This may be related to the nature of care provided in the study setting, which focuses mainly on routine clinical care rather than structured educational or rehabilitative interventions. Therefore, regular follow-up remains important to assess not only whether patients take their medication, but also how they understand, accept, and evaluate medication use. Factors such as illness knowledge, insight, awareness of treatment side effects, and social stigma may also shape patients’ attitudes toward medication and their adherence behavior (19–21).

Non-adherence was common among the studied patients, which is clinically important because poor adherence may increase the risk of relapse and rehospitalization. Although medication non-adherence in psychotic disorders has been widely studied, its associated cognitive and psychosocial factors are still not fully understood. Previous studies have similarly reported poor treatment adherence among patients with psychotic disorders, particularly among patients with schizophrenia and psychotic depression (22, 23). This may be related to illness-related factors, medication side effects, limited insight, stigma, or difficulty maintaining long-term treatment routines. Studies conducted in Turkey have also shown that many patients with schizophrenia do not use their medication regularly and may be readmitted because of non-adherence (24, 25).

The present study also found that the level of perceived social support among patients with psychotic disorders was moderate. This finding is consistent with previous studies reporting that perceived social support among patients with psychotic disorders is often insufficient. Mahmoud et al. (26) reported low social support among two-thirds of patients, while Munikanan et al. (27) found that most patients with schizophrenia had low perceived social support. Similarly, Şahin Altun et al. (23) reported moderate social support levels among patients with schizophrenia.

Patients in the current study reported receiving social support mainly from their families. This may be explained by the fact that most participants were living with family members. Previous national and international studies have also shown that many patients with psychotic disorders live with their families (24, 28). In the cultural context of the present study, family bonds are often strong, and family members commonly play a central caregiving role. This may explain why family support represented the main source of support for many participants.

By contrast, participants reported receiving the least support from a special person in their lives. This may be related to the high proportion of single participants in the current sample. Previous studies have reported that many patients with psychotic disorders are single and may experience difficulty forming or maintaining close personal relationships (23, 24). Social stigma and negative public beliefs about mental illness may also reduce opportunities for close relationships and social connection (10, 22).

The findings showed a significant positive association between perceived social support and medication adherence. Patients who reported higher levels of support tended to report better adherence. Social support may help patients feel accepted, encouraged, and more capable of managing illness-related stress. It may also provide practical assistance, such as reminders, help with appointments, and encouragement to continue treatment. Abd El et al. (22) found that patients from cohesive families and those receiving practical support had better treatment adherence. Similarly, Sendt et al. (29) reported in a systematic review that social support is one of the factors associated with adherence to antipsychotic medication in schizophrenia spectrum disorders. Di Lorenzo et al. (30) also found that being married and having family support may reinforce a more favorable attitude toward medication treatment. In the same line, Şahin Altun et al. (23) highlighted that perceived family support is positively correlated with treatment adherence among patients with schizophrenia.

Although family support in schizophrenia has been described as an important factor in reducing relapse and supporting rehabilitation, less is known about how perceived social and family support is related to the association between subjective cognitive complaints and medication adherence. Therefore, the present findings suggest that perceived social and family support statistically accounted for part of the association between subjective cognitive complaints and medication adherence beliefs. However, because the study used a cross-sectional design, this finding should be interpreted cautiously as an associative statistical finding rather than evidence of causal mediation.

The study found a significant negative association between subjective cognitive complaints and perceived social support, indicating that patients who reported greater self-perceived cognitive difficulties tended to report lower levels of social support. This may suggest that patients who experience more everyday difficulties with attention, planning, memory, or organization may also perceive themselves as less supported or may have more difficulty engaging with available support. Cognitive performance includes several skills needed for planning and carrying out complex daily activities, including attention, concentration, and executive functioning. Executive functions are especially important because they support goal-directed behavior, self-regulation, working memory, mental flexibility, response inhibition, impulse control, and action monitoring (31).

The findings also showed that subjective cognitive complaints were negatively associated with medication adherence. In other words, patients who perceived more frequent cognitive difficulties tended to report poorer adherence. This association may reflect the practical challenges involved in managing medication routines, remembering doses, understanding instructions, planning refills, and maintaining follow-up appointments. Rather than interpreting these findings as evidence that cognitive complaints directly cause non-adherence, the results should be understood as showing that patients’ self-perceived cognitive difficulties are meaningfully related to their reported medication-taking behavior.

According to regression analysis, specific domains of subjective cognitive complaints, particularly executive function and language complaints, were significantly associated with lower medication adherence scores. This suggests that patients who report difficulties in organizing, planning, understanding, or communicating treatment-related information may experience more challenges in maintaining medication routines. Memory complaints showed a marginally significant positive association with medication adherence beliefs, which may reflect the complex and sometimes inconsistent relationship between self-perceived memory difficulties and adherence behavior.

Memory difficulties have been described as an important cognitive concern in schizophrenia because they are related to learning and everyday functioning (32). Difficulties in remembering future intentions may contribute to poor medication management, especially when treatment requires consistent timing and routine (33, 34). Executive functions are also essential for purposeful and goal-directed behavior and may influence patients’ ability to manage complex medication regimens (35). These findings are consistent with previous studies showing that executive function is related to patients’ capacity to manage medication and sustain adherence motivation (36–39).

El-Missiry et al. (40) also reported that memory complaints, particularly digit span and visual memory span, along with executive function deficits, were among the strongest cognitive predictors of medication non-adherence. However, not all studies have reported the same pattern. Jonsdottir et al. (41) found that non-adherent patients with schizophrenia performed better on neurocognitive tests than adherent patients, although the mechanism was unclear. Other studies concluded that neurocognitive deficits were not significantly associated with non-adherence (29, 42). These inconsistent findings may be related to differences in assessment tools, study populations, definitions of adherence, and whether cognition was measured objectively or through patients’ subjective reports.

The indirect-effect analysis indicated that perceived social support statistically accounted for part of the association between subjective cognitive complaints and medication adherence. This suggests that patients who report greater cognitive complaints may also report lower perceived social support, which in turn is associated with poorer adherence. However, given the cross-sectional and self-report nature of the data, this finding should not be interpreted as proof that social support causally mediates this relationship. Instead, it highlights a clinically meaningful pattern of association among subjective cognitive complaints, perceived support, and adherence.

The moderation analysis showed that the interaction between subjective cognitive complaints and perceived social support did not reach statistical significance. This suggests that the buffering role of social support was not clearly supported in the current sample, although the marginal result may warrant further investigation. One possible explanation is that the effect of support may depend more on its quality than its quantity, as not all perceived support is necessarily helpful or encouraging (43). For example, overly controlling or critical family behaviors, even when frequent, may undermine adherence rather than improve it (44). Another explanation is that in cultures where family involvement is common, social support may already be moderately present for many patients, reducing variability and making moderation effects harder to detect (45). Individual differences in insight, stigma perception, and coping style may also shape how social support is experienced and how it relates to adherence behavior (46).

### Strengths & limitation

A key strength of this study is its examination of subjective cognitive complaints, medication adherence, and perceived social and family support within one analytical model among a well-defined outpatient sample of individuals with psychotic disorders. The systematic recruitment process helped reduce selection bias and supported the representativeness of the sample. In addition, the study addresses clinically relevant patient-reported factors that may be useful in psychiatric nursing assessment and adherence support.

However, several limitations should be considered. Because the study used a cross-sectional design, causal relationships cannot be inferred, and the direction of the associations among subjective cognitive complaints, perceived social and family support, and medication adherence cannot be definitively established. Therefore, the indirect-effect findings should be interpreted as statistical evidence that perceived social support accounts for part of the association between subjective cognitive complaints and adherence, rather than as proof of causal mediation. In addition, all measures were self-reported, which may have introduced recall bias, social desirability bias, or shared-method variance. Future research should use longitudinal or interventional designs to clarify temporal relationships and examine whether cognitive compensatory strategies and social support-enhancement programs can improve medication adherence over time.

### Implication

The findings have important implications for psychiatric and mental health nursing practice. Nurses are in a strategic position to identify patients who may be at risk of poor medication adherence because of self-perceived cognitive difficulties or limited perceived social support. Incorporating assessment of subjective cognitive complaints into routine nursing evaluations may help nurses better understand patients’ everyday treatment-management challenges, such as remembering doses, organizing appointments, understanding instructions, and maintaining medication routines.

Nursing interventions should focus on practical adherence support, psychoeducation, and compensatory strategies tailored to patients’ reported cognitive difficulties. These may include medication schedules, reminders, simplified written instructions, pill organizers, follow-up calls, and structured routines. Nurses can also strengthen family and social support by involving caregivers in treatment planning and guiding them to provide support in a way that is encouraging rather than controlling.

From a broader perspective, nursing-led rehabilitation programs may benefit from integrating cognitive compensatory strategies with family psychoeducation and adherence counseling. Training nurses in motivational interviewing, communication skills, and family engagement may further support patients’ ability to sustain medication routines. Such a biopsychosocial nursing approach may help address both individual self-management difficulties and the social context in which adherence behavior occurs.

## Conclusion

This study found that subjective cognitive complaints were significantly associated with medication adherence among patients with psychotic disorders. Higher levels of self-perceived cognitive complaints were associated with lower adherence, while higher perceived social support was associated with better adherence. Perceived social and family support statistically accounted for part of the association between subjective cognitive complaints and medication adherence, although this finding should be interpreted cautiously because of the cross-sectional self-report design.

For nursing practice, the findings highlight the importance of assessing subjective cognitive complaints and perceived social support as part of comprehensive psychiatric care. Psychiatric nurses can support adherence by identifying patients who report cognitive difficulties, providing practical compensatory strategies, and involving family members or caregivers in supportive treatment routines. In conclusion, strengthening nurses’ competencies in cognitive complaint assessment, psychoeducation, adherence counseling, and family involvement may help promote sustained medication adherence and improve outcomes for patients with psychotic disorders.

## Data Availability

The raw data supporting the conclusions of this article will be made available by the authors, without undue reservation.
