# Correction: Associations among subjective cognitive complaints, perceived social support, and medication adherence in patients with psychotic disorders

**DOI:** 10.3389/fpsyt.2026.1993910

**Published:** 2026-10-02

**Authors:** Mohamed Hussein Ramadan Atta, Ahmed Hashem El-Monshed, Nadia Waheed Elzohairy, Ahmed Abdelwahab Ibrahim El-Sayed, Darelglal Ahmed Gassmelseed Abdalla, Mohamed Sayed Abdellatif, Magda Mahmoud Mohammad Elgameel

**Affiliations:** 1 Nursing Department, College of Applied Medical Sciences, Prince Sattam Bin Abdulaziz University, Wadi Addawasir, Saudi Arabia; 2 Department of Nursing, College of Health and Sport Sciences, University of Bahrain, Manama, Bahrain; 3 Department of Psychiatric and Mental Health Nursing, Faculty of Nursing, Mansoura University, Mansoura, Egypt; 4 Faculty of Nursing, Damnhour University, Damanhour, Egypt; 5 Nursing Administration Department, Faculty of Nursing, Alexandria University, Alexandria, Egypt; 6 Department of Nursing Sciences, College of Applied Medical Sciences in Wadi Al-Dawasir, Prince Sattam Bin Abdulaziz University, Alkharj, Saudi Arabia; 7 Department of Psychology, College of Education in Al-Kharj, Prince Sattam Bin Abdulaziz University, Al-Kharj, Saudi Arabia; 8 Department of Nursing, College of Applied Medical Sciences, Prince Sattam Bin Abdulaziz University, Wadi Al-Dawaser, Saudi Arabia

**Keywords:** psychotic disorders, cognitive complaints, medication adherence, social support, sustainability, health, self-assessment, psychiatric care

In the original publication, third author Nadia Waheed Elzohairy’s affiliation was given incorrectly. This should be:

“Faculty of Nursing, Damnhour University, Damanhour, Egypt”

In the original publication, seventh author Magda Mahmoud Mohammad Elgameel’s affiliation was given incorrectly. This should be:

Department of Nursing, College of Applied Medical Sciences, Prince Sattam Bin Abdulaziz University, Wadi Al-Dawaser, Saudi Arabia

In the **Funding** statement, an incorrect grant number was provided for Prince Sattam bin Abdulaziz University. The correct number is PSAU/2025/01/37747.

The correct **Funding** reads:

“The author(s) declared that financial support was received for this work and/or its publication. The authors extend their appreciation to Prince Sattam bin Abdulaziz University for funding this research work through the project number (PSAU/2025/01/37747).”

A correction has been made to the Keywords. The following keywords were missing from the original article: “sustainability, health”. The list has also been reordered. The final list of keywords reads as follows:

“psychotic disorders, cognitive complaints, medication adherence, social support, sustainability, health, self-assessment, psychiatric care”

The original version of this article has been updated.

